# Sevoflurane induces inflammation in primary hippocampal neurons by regulating Hoxa5/Gm5106/miR-27b-3p positive feedback loop

**DOI:** 10.1080/21655979.2021.2005927

**Published:** 2021-12-24

**Authors:** Zifu Zhu, Li Ma

**Affiliations:** a Huizhou Municipal Central Hospital, Huizhou, Guangdong, PR China; b First Affiliated Hospital of Kunming Medical University, Kunming, Yunnan, PR China

**Keywords:** Sevoflurane, inflammation, Gm5106, Hoxa5, miR-27b-3p

## Abstract

Postoperative cognitive dysfunction (POCD) is a normal condition that develops after surgery with anesthesia, leading to deterioration of cognitive functions. However, the mechanism of POCD still remains unknown. To elucidate the POCD molecular mechanism, sevoflurane was employed in the present study to generate neuroinflammation mice model. Sevoflurane treatment caused inflammatory markers IL6, IL-10 and TNF-α high expression in primary hippocampal neurons and blood samples. Long non-coding RNA Gm5106 was found to be increased after being stimulated with sevoflurane. Silencing Gm5106 inhibited neuron inflammation. In the meanwhile, Gm5106 was identified as a direct target of miR-27b-3p that was inhibited by sevoflurane and related to inflammation suppression. In addition, transcription factor (TF) Hoxa5 was validated to activate Gm5106 through two binding motifs in the promoter region after sevoflurane exposure. Furthermore, miR-27b-3p also directly targeted Hoxa5 3ʹUTR, which affected nuclear Hoxa5 protein served as TF. Hoxa5 protein instead of 3ʹUTR reduced miR-27b-3p, in which Gm5106 knocking down abrogated this effect. In conclusion, sevoflurane induces neuroinflammation through increasing long non-coding RNA Gm5106, which is transcriptionally activated by Hoxa5 and directly targeted by miR-27-3p. Apart from that, Hoxa5, Gm5106, and miR-27b-3p form a positive feedback loop in sevoflurane stimulation.

## Introduction

Postoperative cognitive dysfunction (POCD), which is also named abnormal cognition after anesthesia, remains a common complication following anesthesia [1]. POCD is frequently characterized as symptoms such as dysfunctions in cognitive function containing memory and executive functions [1]. Clinical data indicate that POCD incidence ranges from 19.2% to 25.8% after 1 week of surgery and reaches 9.9% after 3 weeks [2,3]. Compound from the fungus exhibits a protective effect in POCD [4]. However, the detailed mechanism is still uncovered, which becomes an obstacle for drug designing and POCD prevention.

Neuroinflammation is a main cause of POCD, which has been clarified in recent studies [5]. Elevated interleukins and cytokines could be observed in cognitive decline. Preclinical studies supported that neuroinflammation is a potential pathogenic mechanism of POCD. In some cases, mesenchymal stem cell-derived extracellular vesicles attenuate elevated neuroinflammation [6]. Sevoflurane is an inhalational anesthetic with common application in surgery. Studies have shown that sevoflurane affects cancer progression and inflammations via mediating signaling pathways and gene expressions [7,8]. It altered pathophysiological situations of brain, which leads to neuroinflammation and results in neurodegenerative changes. Nevertheless, the detail molecular mechanism of the effect of sevoflurane on neuroinflammation and POCD still remains unclear. Therefore, it is important to clarify the underlying molecular mechanism of neuroinflammation caused by sevoflurane to avoid the side effect.

Long non-coding RNAs (lncRNAs) are RNAs longer than 200 nt and lack protein coding abilities [9]. Accumulating evidences illustrate that lncRNAs participate in the development of human diseases containing cancer progression [10], inflammatory responses [11], and chemotherapy resistance [12]. In POCD, several lncRNAs were found to be dysregulated. LncRNA PCAI knocking down inhibited cell death rates and attenuated inflammation through regulating SUZ12 in POCD [13]. Both uc009qbj.1 and ENSMUST0000017438 were confirmed to be positively associated with inflammation and apoptosis, which suggested that lncRNAs exert potential key roles in mediating neuronal inflammation [14]. Additionally, lncRNAs function as a brake, which can inhibit neuron inflammation in POCD. The overexpression of lncRNA Gm43050 alleviated apoptosis and inflammation response induced by sevoflurane treatment by regulating miR-640/ZFP91 [15]. Our current research proved that the lncRNA Gm5106 was overexpressed in sevoflurane stimulated neuroinflammation mice model with high inflammatory responses. Bioinformatic analysis revealed that Hoxa5 functioned as a transcription factor of Gm5106, which controlled Gm5106 expression followed by sevoflurane exposure.

In addition, microRNA regulations were also considered in the present study. It is hypothesized that sevoflurane induces inflammation through inducing Gm5106 that is transcriptionally activated by Hoxa5 and interacts with miR-27b-3p. Actually, miR-27b-3p was identified to alleviate neuroinflammation via different regulations. Several genes such as PPARγ, STING and TGF-β are targets of miR-27b-3p and are involved in neuroinflammation response [16,17]. HOXA5 also contributes to neuroinflammation through interacting with TGF signaling pathway [18].

In this study, the possible positive feedback loop of Hoxa5/Gm5106/miR-27b-3p was elucidated. Our findings revealed a possible new target/biomarkers for preventing POCD and protecting neurons from damages after anesthesia with sevoflurane.

## Material and methods

### Animal model with sevoflurane treatment

**Figure 1.**
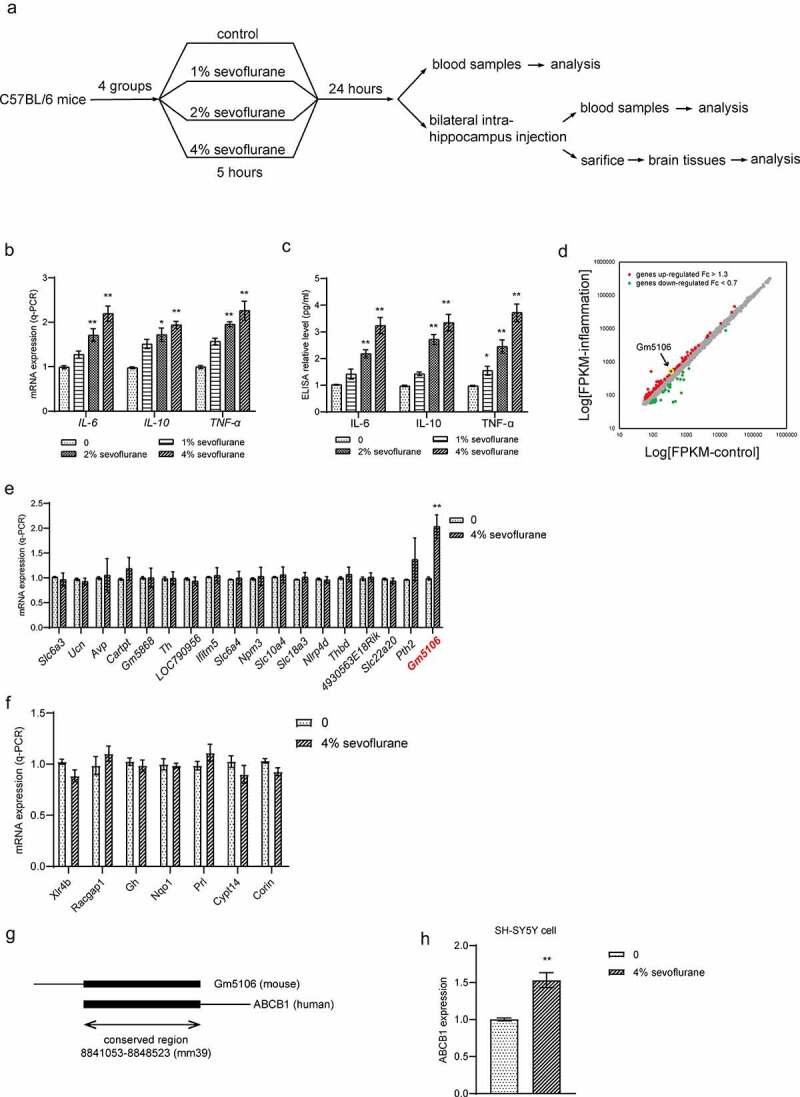
Differential gene expression in primary hippocampal neuron inflammation caused by sevoflurane. (a) The flow chart of animal experiment. (b) Inflammatory markers were measured by q-PCR analysis in hippocampus of mice treated by sevoflurane with different concentrations. The results were compared to group 0. (c) Inflammatory markers in mice blood were tested by ELISA assay after treated by sevoflurane with different concentrations. The results were compared to group 0. (d) Differential gene expression in primary hippocampal neuron inflammation. Up-regulated genes with fold change over 1.3 were marked as red dots. Down-regulated genes with fold change less than 0.7 were marked as green dots. (e) 18 top up-regulated genes were validated by q-PCR with or without sevoflurane treatment in mice hippocampus. The results were compared to group 0. (f) 7 top down-regulated genes were validated by q-PCR with or without sevoflurane treatment in mice hippocampus. The results were compared to group 0. (g) Scheme of conservation between Gm5106 and ABCB1. (h) q-PCR analysis of ABCB1 expression with 4% sevoflurane treatment in human SH-SY5Y cell. *p < 0.05, **p < 0.01

Total 24 healthy male C57BL/6 mice (age range: 4–6 weeks, body weight: 18–22 g) were purchased from Shanghai Laboratory Animal Research Center of the Chinese Academy of Sciences for neuroinflammation mice model establishment. Mice were randomly divided into four groups including control group and sevoflurane groups with different concentrations. In detail, in control group, mice only inhaled air containing 30% O_2_ (0%). In sevoflurane treatment groups, mice were kept in anesthesia chamber (50 cm × 35 cm × 35 cm, one mouse per chamber) and inhaled with air containing 30% O_2_ with 1%, 2% and 4% sevoflurane (Abbott Ireland Ltd., Dublin, Ireland), respectively, for 5 h. The chamber was kept at 37°C and the activity of mice was observed continuously. After 24 h of anesthesia, blood samples were collected and then all mice were sacrificed and the brain tissue was dissected. Mice hippocampus was removed and lysed by RIPA lysis buffer (Beyotine, China) for Western blot analysis or by Trizol (Qiagen, Germany) for q-PCR analysis. In groups with RNA oligo and vector treatments, oligos or vector solutions were prepared in saline at the concentration of 5 μg/μl. Before the bilateral intrahippocampus injections and sacrifice, mice were anesthetized by sodium pentobarbital (50 mg/kg) via intraperitoneal injection. Then bilateral intrahippocampus injections were performed in the hippocampal CA1 region. The ideal position is 3.0 mm posterior to the bregma, 2.2 mm lateral to the midline, and 3.5 mm beneath the surface of the skull according to previous study [19]. 2 μl of oligos or vectors were injected. The flow chart of animal experiment is presented in Figure 1a. The animal experiments were reviewed and approved by the Ethics Committee of Kunming Medical University.

### Cell culture

Primary hippocampal neurons were derived and cultured from mice as described previously [20] and cultivated in Neurobasal™ Medium (Gibco, USA) filled with 10% fetal bovine serum (FBS) and B-27™ Supplement (Gibco, USA). Human SH-SY5Y cells (CRL-2266) were obtained from ATCC and maintained in DMEM medium with 10% FBS. Cells were treated with sevoflurane at the concentration of 0%, 1%, 2%, and 4% for 6 h before further analysis.

### Vectors, siRNAs, and transfection

Gm5106 siRNA, Hoxa5 siRNA pools and miRNA mimics used in this study were purchased from QIAGEN (Germany) and transfected into cells with Lipofectamine 2000 (Thermo Fisher, USA) according to protocol provided by the manufacturer when seeded cells reached over 75% confluence. The final concentration of siRNA or miRNA was 25 nM [21]. After transfection, cells were incubated for 48 h and then analyzed by continued assays. The Gm5106 siRNA sequence information is listed in Table 1. For overexpression vector transfection, all procedures were same with siRNA and miRNA except the concentration was used. In 6-well plate, 2.5 μg vector was added per well. Hoxa5 overexpression was performed through pcDNA3.1(+) vector with HA tag and named with pcDNA3.1-Hoxa5-HA. The vector construction was accomplished by Beijing Genomics Institute (BGI).

**Table 1.** The sequence information of siGm5106 (5ʹ-3ʹ)

siRNA	Sequence
siGm5106	GGAUGAUAGUCCUAUACAAUU

### Quantitative real-time PCR (q-PCR)

Total RNAs were extracted and purified from treated cells with High Pure RNA Isolation Kit (Roche, Switzerland). Reverse transcription of RNA was performed using a PrimeScript™ RT reagent kit with random oligo dT primers. Q-PCR was conducted with SYBR® Premix Ex Taq™ (RR420A; Takara). The data were normalized to GAPDH and calculated using 2^-ΔΔCt^ value [22]. MiR-27-3p mature miRNA primers were purchased from QIAGEN (Germany). Other primers used are listed in Table 2.

**Table 2.** Primers used in q-PCR. (5ʹ to 3ʹ)

Genes	Primers (5ʹ to 3ʹ)
IL-6 F	CTGCAAGAGACTTCCATCCAG
IL-6 R	AGTGGTATAGACAGGTCTGTTGG
IL-10 F	GCTGGACAACATACTGCTAACC
IL-10 R	ATTTCCGATAAGGCTTGGCAA
TNF-α F	CTGAACTTCGGGGTGATCGG
TNF-α R	GGCTTGTCACTCGAATTTTGAGA
Gm5106 F	TCCGCCCTGGCGCGCTCC
Gm5106 R	TGTCATTTCACAGTTGAGCGACTT
Hoxa5 F	CTCATTTTGCGGTCGCTATCC
Hoxa5 R	ATCCATGCCATTGTAGCCGTA

### Western blot

Total protein was isolated from primary hippocampal neurons after different treatments by applying RIPA lysis buffer. Protein concentrations of each group were quantified with BCA kit based on instructions. Total 30 μg protein was loaded on 10% sodium dodecyl sulfate polyacrylamide gel. The electrophoresis conditions were 120 V for120 min. Then transferred to polyvinylidene difluoride membrane (300 mA, 90 min). Membranes were blocked by 5% milk in 1xTBST and then incubated with primary antibodies Hoxa5 (1:1000, Abcam, USA), β-actin (1:2000, Abcam, USA) overnight at 4°C. After incubation, membranes were washed for 3 times with TBST and then incubated with the secondary antibody diluted in 5% milk at the ratio 1:10000.

### Enzyme linked immunosorbent assay (ELISA)

Blood samples used for ELISA were collected and prepared according to the protocol given by the ELISA kit manufacturer. The IL-6 (ab178013), IL-10 (ab255729) and TNF-α (ab208348) were quantified using enzyme linked immunosorbent assay (ELISA) assay strictly following the instructions of the product.

### ChIP-qPCR (q-ChIP)

Q-ChIP was performed followed the datasheets of iDeal ChIP-qPCR Kit (Diagenode, USA). Hoxa5 was ectopically overexpressed in a tool cell-line HEK293, and then chrome DNA was isolated through Hoxa5 antibody precipitation. Chrome DNA was sonicated for 30 min to get DNA fragments with appropriate size (400–600 bp). The expression of Gm5106 was evaluated by q-PCR.

### Dual luciferase assay

The 3ʹ- untranslated regions (3ʹUTRs) of Gm5106 and Hoxa5 containing wild type (wt) or mutant (mut) binding sites for miR-27b-3p were cloned into pGL3 control vector. For transcription factor validation, the mutation strategy is shown in Figure 3d and all promoter regions were cloned into pGL3 control vector. Luciferase activity of each group transfected with different vectors were measured by microplate reader and analyzed based on the protocol of the instruction of the kit.

**Figure 2.**
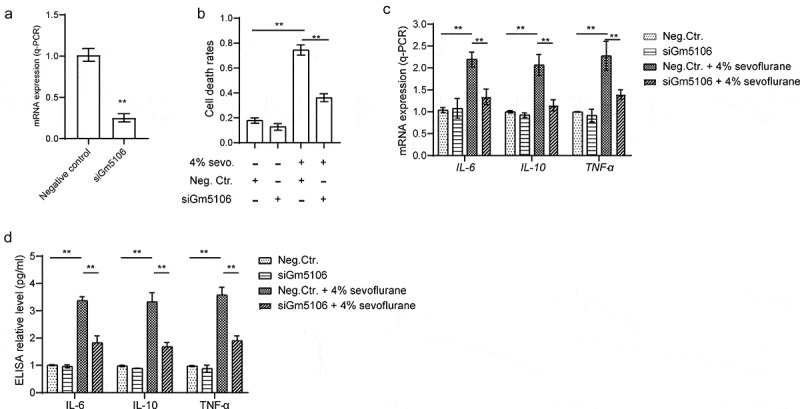
Gm5106 related to primary hippocampal neuron inflammation caused by sevoflurane. (a) Gm5106 was silenced by specific siRNA and tested by q-PCR in derived neuron. (b) Cell death rates were measured by Trypan Blue staining after treated with 4% sevoflurane or Gm5106 siRNA in neuron. (c) Inflammatory markers were measured after neuron treated by 4% sevoflurane with Gm5106 siRNA (siGm5106) or a negative control (Neg. Ctr.) in hippocampus of mice. (d) Inflammatory markers in blood samples were measured after mice treated by 4% sevoflurane with Gm5106 siRNA (siGm5106) or a negative control (Neg. Ctr.). **p < 0.01

**Figure 3.**
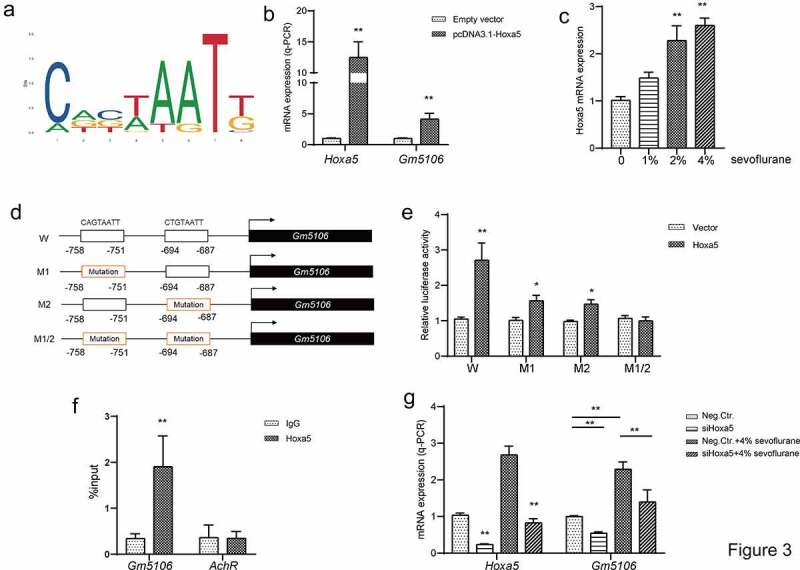
Hoxa5 transcriptionally activated Gm5106. (a) Hoxa5 binding motif was predicted through JASPAR. (b) In HEK-293 T cells, Hoxa5 was overexpressed by pcDNA3.1-Hoxa5 vector and the effect on Gm5106 expression was measured. (c) The effect of sevoflurane on Hoxa5 expression was tested in mice model. (d) The mutation strategy was designed to confirm transcription relations. Two putative binding sites of Hoxa5 in Gm5106 promoter region were mutated and named by M1, M2 and M1/2. (e) Luciferase reporter vectors containing Gm5106 wild type or mutant promoter region were tested. (f) The binding between Hoxa5 and Gm5106 was confirmed by q-ChIP assay, AchR was served as a negative control. (g) The effect of Hoxa5 siRNA on Gm5106 expression with or without 4% sevoflurane treatment was measured. *p < 0.05, **p < 0.01

### Trypan blue staining

After different treatments, cells were digested with Trypsin (Gibco, USA) and made single-cell suspension in medium. Then centrifuge was conducted at 1000 rpm for 2 min. Resuspended cells in fresh medium. Added 0.4% Trypan blue solution into cell suspension to get final concentration 0.04%. Cells were counted in 3 min and the dead cells were stained blue.

### Statistics

SPSS 21.0 was used to calculate all the values (means ± standard error of the mean). Statistical analyses were analyzed with Student’s t-test in two groups comparison. One-way analysis of variance (ANOVA) test was used to verify the significance among experimental groups. The statistical significance was p < 0.05.

## Results

### LncRNA Gm5106 is highly expressed in primary hippocampal neuron inflammation caused by sevoflurane

Neuroinflammation is mostly caused by POCD. In mice models, increased interleukins in mouse hippocampus are associated with cognitive failure. In surgical patients with cognitive decline, elevations of pro-inflammatory cytokines were observed in central nervous system and the systemic circulation. Additionally, recent studies also showed that sevoflurane induced POCD development through changing inflammation response. Therefore, our study employed the neuroinflammation mice model stimulated by sevoflurane to clarify the underlying mechanisms. To validate the effect of sevoflurane on primary hippocampal neuron, we generated neuroinflammation mice model stimulated by sevoflurane with different concentrations (0%, 1%, 2% and 4%). Both q-PCR (Figure 1b) on dissected hippocampus and ELISA on blood samples (Figure 1c) indicated that inflammatory markers such as IL-6, IL-10 and TNF-α were increased with sevoflurane exposure. Notably, the effect of sevoflurane was concentration dependent. Then, to obtain the differentially expressed genes in primary hippocampal neuron inflammation, we analyzed gene expression profiles of GSE106620. A total of 120 genes were identified to be highly expressed over 1.3 times while 36 genes were down regulated less than 0.7 times in neuron with inflammation (Figure 1d). Subsequently, we performed q-PCR analysis on differentially expressed genes with change over 1.5 times or less than 0.5 times. Unexpectedly, 4% sevoflurane inhalation slightly altered predicted down-regulated genes in dissected hippocampus. Several predicted up-regulated genes were induced by sevoflurane, where the lncRNA Gm5106 was induced over 2.0-fold change (Figure 1e and f). Therefore, Gm5106 was a promising target of sevoflurane in inflammation stimulation. To confirm the possibility of further translational research of GM5106 in human cells or tissues, we evaluated the conservation of Gm5106 in human genome. Gm5106 exhibited high conservation with human gene ABCB1 (Figure 1g). Since previous studies have identified that ABCB1 is positively related to inflammation and drug resistance [23], it is possible that sevoflurane could stimulate human neuron inflammation by inducing ABCB1. In fact, ABCB1 was induced in human SH-SY5Y cells treated with 4% sevoflurane (Figure 1h). The above-mentioned results suggested that sevoflurane induced inflammation through up-regulating Gm5106 in primary hippocampal neuroinflammation mice model.

### The effect of Gm5106 on neuroinflammation

Next, the effect of Gm5106 on promoting neuroinflammation was investigated. Initially, the function of Gm5106 on cell death was evaluated. According to the results, cell death rate was remarkably reduced by silencing Gm5106 with siRNAs (siGm5106) in derived neurons treated with 4% sevoflurane (Figure 2a and b). However, silencing Gm5106 slightly impacted on cell death of neurons without receiving sevoflurane treatment (Figure 2b). In addition, inflammatory markers were tested after Gm5106 was knocked down in mice model. As illustrated in Figure 2c and d, inflammatory markers including IL-6, IL-10 and TNF-α were suppressed by siGm5106 in sevoflurane treatment group. However, the changes of Gm5106 level slightly influenced inflammatory markers in dissected hippocampus and blood without sevoflurane stimulation. As a result, Gm5106 mediated the effect of sevoflurane on neuroinflammation.

### Sevoflurane induces Hoxa5 to transcriptionally activate Gm5106

To further investigate the regulatory axis associated with Gm5106, the possible upstream regulators were searched. Based on JASPAR and UCSC genome browser, two Hoxa5 binding motifs CAGTAATT and CTGTAATT were discovered in Gm5106 promoter region (−2000 ~ 0 bp) in JASPAR (Figure 3a). Q-PCR results indicated that Gm5106 was induced by Hoxa5 ectopic expression in derived primary hippocampal neuron (Figure 3b). Interestingly, Hoxa5 was also induced by sevoflurane treatment (Figure 3c) in mice model. The target between Hoxa5 and Gm5106 was validated by introducing mutations as the strategy shown in Figure 3d. Dual-luciferase assay demonstrated that both two binding sites were essential for Hoxa5 transcription function (Figure 3e). Furthermore, q-ChIP assay also demonstrated that Gm5106 was enriched after Hoxa5 overexpression (Figure 3f) in cell. Furthermore, silencing Hoxa5 by a specific siRNA reversed the effect of sevoflurane on Gm5106 in mice model (Figure 3g). Collectively, Gm5106 is a direct target of Hoxa5. Sevoflurane induced Gm5106 expression through Hoxa5 function.

### MiR-27b-3p interacts and directly targets Gm5106

**Figure 4.**
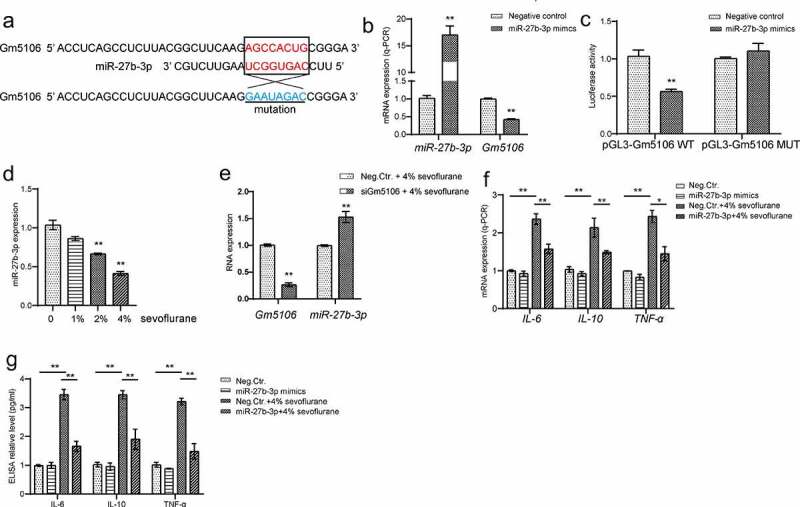
Gm5106 interacted with miR-27b-3p. (a) Gm5106 was predicted as a direct target of miR-27b-3p. (b) The effect of miR-27b-3p mimics on Gm5106 expression was tested by q-PCR. (c) Target between Gm5106 and miR-27b-3p was confirmed by luciferase assay. (d) The effect of sevoflurane on miR-27-3p expression was tested by q-PCR in mice model. (e) The effect of siGm5106 on miR-27b-3p expression in mice with sevoflurane treatment. (f) Inflammatory markers were measured after mice treated by 4% sevoflurane with miR-27b-3p mimics or negative control (Neg. Ctr.). (g) Inflammatory markers in blood samples were measured after neuron treated by 4% sevoflurane with miR-27b-3p mimics or negative control (Neg. Ctr.). *p < 0.05, **p < 0.01

Since previous studies have uncovered that miRNA is one of main regulator of lncRNA via direct targeting, the potential involved miRNA was investigated in this study. From bioinformatics tool ENCORI analysis, miR-27b-3p was characterized to target Gm5106 (Figure 4a). Indeed, Gm5106 was repressed by ectopic miR-27b-3p expression in derived primary hippocampal neuron (Figure 4b). Based on dual-luciferase reporter assay results, the luciferase activity of wild type Gm5106 reporter vector was significantly suppressed when co-transfected with miR-27b-3p mimics in comparison with the control group and mutant Gm5106 group (Figure 4c). Interestingly, sevoflurane stimulation repressed miR-27b-3p expression in mice model (Figure 4d), suggesting a potential function of miR-27b-3p in sevoflurane and Gm5106 regulation. Additionally, Gm5106 silencing induced miR-27b-3p expression in neurons with sevoflurane treatment (Figure 4e), implying that Gm5106 and miR-27-3p interacted with each other. Furthermore, ectopic miR-27b-3p expression inhibited inflammatory markers in mice with 4% sevoflurane treatment (Figure 4f and g). Therefore, miR-27-3p is an anti-inflammation miRNA and participates sevoflurane-Gm5106 regulation.

### Sevoflurane induces inflammation through Hoxa5/Gm5106/miR-27-3p positive feedback loop

**Figure 5.**
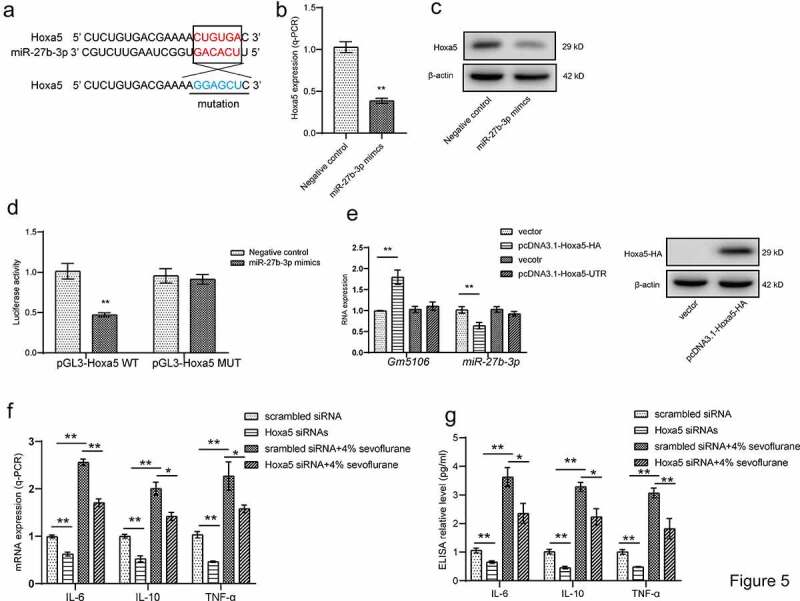
Hoxa5/Gm5106/miR-27-3p positive feedback loop. (a) Hoxa5 was predicted as a direct target of miR-27b-3p. (b) The effect of miR-27b-3p mimics on Hoxa5 expression was tested by q-PCR. (c) The effect of miR-27b-3p mimics on nuclear Hoxa5 protein expression was tested by Western blot. (d) Target between Hoxa5 and miR-27b-3p was confirmed by luciferase assay. (e) Hoxa5 protein and 3ʹUTR overexpression affected Gm5106 and miR-27b-3p expression. (f) Inflammatory markers were measured after neuron treated by 4% sevoflurane with Hoxa5 siRNAs or negative control (scrambled siRNA). (g) Inflammatory markers were measured by ELISA in cell medium after neuron treated by 4% sevoflurane with Hoxa5 siRNAs or negative control (scrambled siRNA). *p < 0.05, **p < 0.01

Since Hoxa5 was also featured as a direct target of miR-27b-3p through ENCORI online software (Figure 5a), we also explored that the possibility of positive feedback loop formed by Hoxa5, Gm5106 and miR-27b-3p. Hoxa5 was repressed by ectopic miR-27b-3p in both mRNA and protein level in derived primary hippocampal neuron (Figure 5b and c). The dual-luciferase reporter assay presented that miR-27b-3p mimics significantly inhibited luciferase activity of wild-type Hoxa5 reporter vector compared with the control group and mutant group (Figure 5d), demonstrating that Hoxa5 is a direct target of miR-27b-3p. Furthermore, this study performed analysis on miR-27b-3p and Gm5106 expression by introducing different Hoxa5 ectopic expression vectors in neuron. Interestingly, the expressions of miR-27b-3p and Gm5106 were altered by the vector expressing Hoxa5 protein. However, Hoxa5 3ʹUTR was inessential for miR-27b-3p and Gm5106 regulation (Figure 5e). In summary, Hoxa5 is a direct target of miR-27b-3p. In addition, silencing Hoxa5 in primary hippocampal neuron partly reversed the effect of 4% sevoflurane treatment on inducing inflammatory markers (Figure 5f and g), tested by q-PCR and ELISA assays. Interestingly, the down-regulation of Hoxa5 repressed the expression of inflammatory markers with or without sevoflurane treatment. Above results suggest that Hoxa5 is critical mediator in neuroinflammation. Taken together, Hoxa5-Gm5106-miR-27b-3p form a positive feedback loop, which mediates the pro-inflammation effect of sevoflurane.

## Discussion

Postoperative cognitive dysfunction (POCD) normally occurs after surgery with anesthesia, causing deterioration of cognitive functions [1,3]. Basically, inflammation responses were found to be activated after anesthesia, in which a group of inflammatory factors were induced, leading to neuron damage. Accumulating evidences have revealed that long non-coding RNA exerts an important role in neuron inflammation. In our study, Gm5106 was selected from GEO datasets. We confirmed that Gm5106 was up-regulated by sevoflurane stimulation. In addition, several inflammatory markers including IL-6, IL-10 and TNF-α were observed to be repressed when Gm5106 was silenced after 4% sevoflurane treatment. Obviously, Gm5106 slightly affected inflammatory markers in neuron without sevoflurane exposure. Gm5106 is induced by sevoflurane and is a promising target of preventing POCD caused by sevoflurane. Furthermore, we compared the conservation between Gm5106 and human genome. An inflammation-related gene ABCB1 (alternatively termed MDR1) was found to be highly conserved with Gm5106, implying the possibility that sevoflurane induces inflammation through ABCB1 in human. ABCB1 plays vital roles in nerve systems. ABCB1 participates in the regulation of various pathological conditions including inflammation, heat shock and stroke [24]. Therefore, altered Gm5106 by sevoflurane represents ABCB1 as a biomarker in human. Sevoflurane treatment-induced ABCB1 expression in human SH-SY5Y cells, whereas the detailed regulation between sevoflurane and ABCB1 still needs further investigation in human.

Furthermore, Hoxa5 was identified as a transcription factor of Gm5106. There were two Hoxa5 binding motifs (CAGTAATT and CTGTAATT) in promoter region of Gm5106. Both two motifs were required for Hoxa5 transcription ability. Hoxa5 usually plays tumor promotion roles in severe diseases such as breast cancer [25], acute myeloid leukemia [26] and colorectal cancer [27]. Notably, HOXA5 is also correlated to immune response involving M2 macrophage and TGF-β signaling pathways [28]. It is promising to investigate the effect of sevoflurane on immune system via HOXA5 connection, which will provide a deep explanation on neuroinflammation caused by sevoflurane. In the present study, sevoflurane was verified to increase Hoxa5, which resulted in an increased Gm5106 level. Consequently, Hoxa5 is a critical regulator between sevoflurane and Gm5106 in neuron inflammation. However, the target between HOXA5 and human ABCB1 is still needed to be validated in human cells.

**Figure 6.**
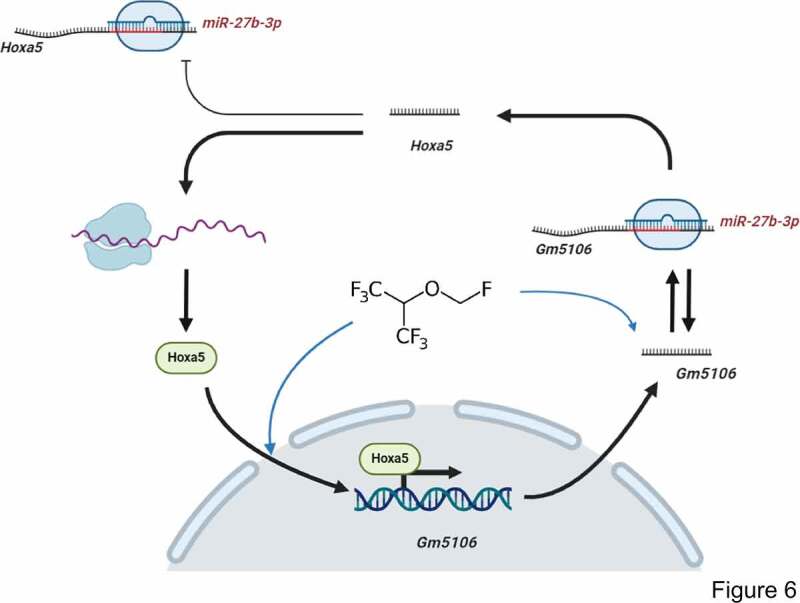
Regulation module of the effect of sevoflurane on neuron inflammation. Sevoflurane induced both long non-coding RNA Gm5106 and TF Hoxa5. During the regulation, Hoxa5 transcriptionally activates Gm5106 which is a direct target of miR-27b-3p. In cytoplasm, Gm5106 interacts with miR-27b-3p and leads to less inhibition of Hoxa5 by miR-27b-3p. Increased Hoxa5 translocates into nucleus to activate Gm5106 and forms positive feedback loop

MicroRNAs are non-coding RNAs with 18–22 nucleotides and target 3ʹUTR of mRNAs [29]. Previous studies have identified that miRNAs take part in lncRNA function through directly targeting or competitive endogenous RNA networks [29]. In our study, Gm5106 was found to be a direct target of miR-27b-3p. Interestingly, Gm5106 also regulated miR-27b-3p through seed sequence sponging. In previous studies, miR-27b-3p showed an inhibitory effect on inflammation [30] and cancer progression [31]. Thus, our study further supports that miR-27b-3p is a promising biomarker in POCD. Furthermore, we also clarified that miR-27b-3p directly targeted Hoxa5, implying a potential regulation axis among Hoxa5, Gm5106 and miR-27b-3p. Notably, only Hoxa5 protein ectopic expression induced Gm5106 and repressed miR-27b-3p, indicating that Hoxa5 lacks the ability to increase miR-27b-3p by RNA-RNA sponging. The underlying mechanism refers to that Hoxa5 activates Gm5106, which results in a decreased miR-27b-3p level by Gm5106/miR-27b-3p sponging. The mechanism is summarized in Figure 6.

## Conclusion

Sevoflurane induces neuroinflammation through increasing long non-coding RNA Gm5106 expression, which is transcriptionally activated by Hoxa5 and directly targeted by miR-27-3p. Hoxa5, Gm5106 and miR-27b-3p form a positive feedback loop in sevoflurane stimulated inflammation. However, this study still lack the evidence of HOXA5-ABCB1 in human samples. In current stage, miR-27-3p. Hoxa5 and Gm5106 are supposed to serve as biomarkers of POCD induced by sevoflurane.

## Data Availability

The authors confirm that the data supporting the findings of this study are available within the article.
